# Cell-Adhesion Properties of β-Subunits in the Regulation of Cardiomyocyte Sodium Channels

**DOI:** 10.3390/biom10070989

**Published:** 2020-07-01

**Authors:** Samantha C. Salvage, Christopher L.-H. Huang, Antony P. Jackson

**Affiliations:** 1 Department of Biochemistry, University of Cambridge, Cambridge CB2 1QW, UK; clh11@cam.ac.uk; 2 Department of Physiology, Development and Neuroscience, University of Cambridge, Cambridge CB2 3EG, UK

**Keywords:** voltage-gated sodium (Nav) channels, Nav1.5, sodium (Nav) channel β-subunits, cell-adhesion, ephaptic conduction

## Abstract

Voltage-gated sodium (Nav) channels drive the rising phase of the action potential, essential for electrical signalling in nerves and muscles. The Nav channel α-subunit contains the ion-selective pore. In the cardiomyocyte, Nav1.5 is the main Nav channel α-subunit isoform, with a smaller expression of neuronal Nav channels. Four distinct regulatory β-subunits (β1–4) bind to the Nav channel α-subunits. Previous work has emphasised the β-subunits as direct Nav channel gating modulators. However, there is now increasing appreciation of additional roles played by these subunits. In this review, we focus on β-subunits as homophilic and heterophilic cell-adhesion molecules and the implications for cardiomyocyte function. Based on recent cryogenic electron microscopy (cryo-EM) data, we suggest that the β-subunits interact with Nav1.5 in a different way from their binding to other Nav channel isoforms. We believe this feature may facilitate *trans*-cell-adhesion between β1-associated Nav1.5 subunits on the intercalated disc and promote ephaptic conduction between cardiomyocytes.

## 1. Introduction

**Figure 1:**
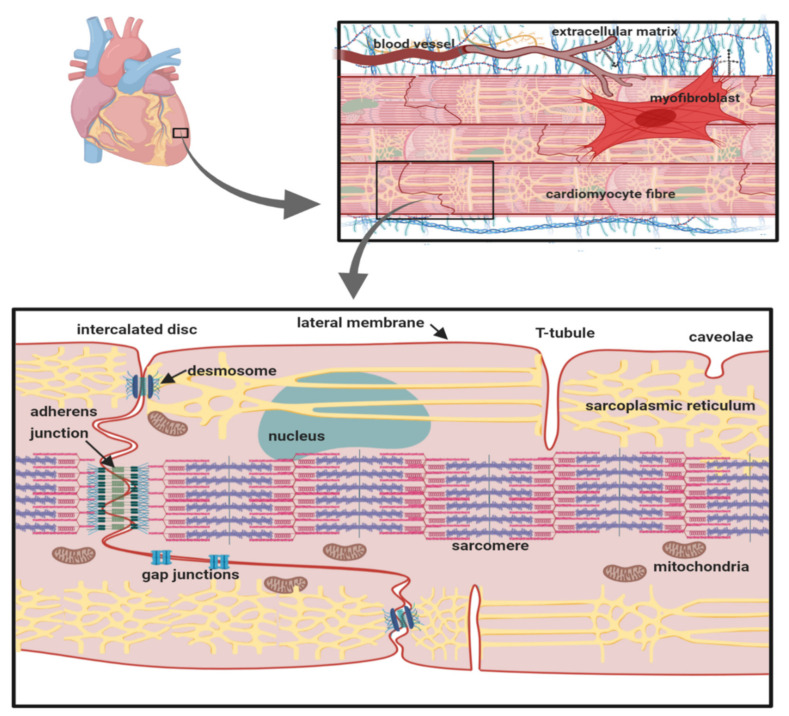
The cardiomyocyte: its anatomical and cellular context. The location of key organelles, membrane compartments and molecular components mentioned in the text are indicated.

Cardiomyocytes within cardiac muscle bundles perform the involuntary contraction and relaxation cycle that is the cellular basis of the heartbeat. Cardiomyocytes possess unique adaptations to ensure this process is tightly synchronised between individual cells. In particular, the cardiomyocytes are both physically and electrically connected to each other via their intercalated discs. On the lateral membrane, T-tubules facilitate the transmission of the electrical signal from the cell surface, to deeper within the cell. This stimulates the release of calcium from the sarcoplasmic reticulum and the initiation of sarcomere contraction (Figure 1) [1].

The cardiac action potential underlies electrical signalling and is initiated by the transient depolarisation of voltage-gated sodium (Nav) channels (for further details, see Ref. [2], this volume). The Nav channel α-subunit (Mwt ~220–250 kDa) contains the ion-selective pore. In the human genome, there are nine different functional Nav channel α-subunit genes encoding proteins Nav1.1-1.9. Different Nav channel α-subunit isoforms are expressed in a tissue-specific manner and exhibit distinct gating behaviour, presumably tailored to their physiological context. In the cardiomyocyte, Nav channels with different gating properties can also be correlated with their differing sub-cellular localisation. The major Nav channel isoform expressed in the heart is Nav1.5. It is mainly localised at the intercalated disc and within caveolae on the sarcolemmal lateral membrane [3]. Cardiomyocytes also express smaller amounts of the neuronal channels Nav1.1, Nav1.3 and Nav1.6, which are predominantly localised in the T-tubules [4,5]. This pattern is striking and is likely to be functionally significant. For example, on a given cardiomyocyte, all Nav channels will experience the same resting potential. However, Nav1.5 activates at more negative potentials and more slowly compared to neuronal Nav channels. Thus, Nav1.5 at the intercalated disc and on the sarcolemma may initiate the cardiac action potential as it propagates from one cardiomyocyte to another within the muscle fibre [5,6]. By contrast, a delayed T-tubular excitation of the neuronal Nav channels will be matched by their more negative threshold for excitation and the more rapid kinetics of activation. This, combined with the close structural association between the neuronal Nav channels, the sodium-calcium exchanger (NCX) and the voltage-gated calcium channels on the T-tubular membrane and with the ryanodine receptors (RyR) on the adjacent sarcoplasmic reticulum, permits T-tubular activation that is synchronous with the surface action potential and that optimally initiates excitation-contraction coupling [4,7].

### 1.1. The Nav Channel α-Subunit

**Figure 2:**
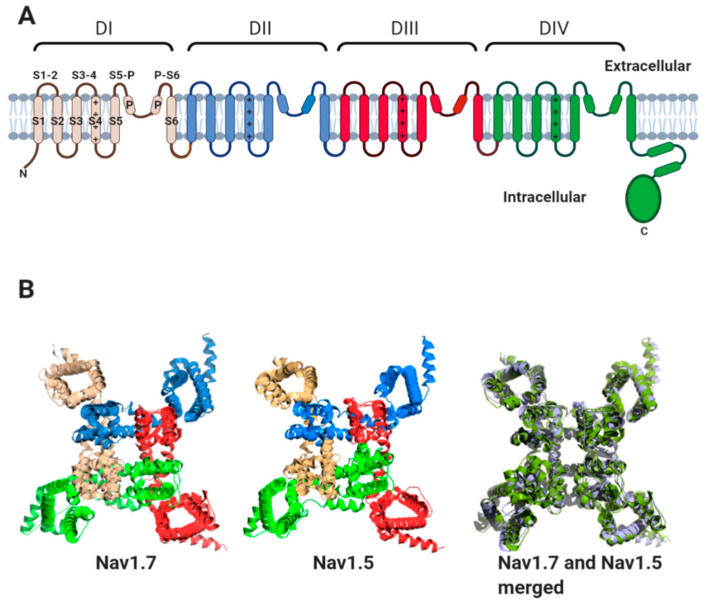
The Nav channel α-subunit. (**A**) Cartoon representation showing internally homologous domains DI-DIV. In DI, the location of transmembrane alpha-helices, S1–S6, the extracellular loops (S1-2; S3-4; S5-P and P-S6) and the re-entrant P helices are indicated. The positive charges on the S4 helices of each domain are indicated. (**B**) Three-dimensional structures of human Nav1.7 (PDB: 6JH8I), rat Nav1.5 (PDB: 6UZ3), and their aligned structures. The channels are viewed from above the plane of the plasma membrane. For Nav1.5 and Nav1.7, the domains DI-DIV are coloured as in (**A**). For aligned structures, Nav1.7 is coloured blue–white and Nav1.5 is coloured pale green.

All Nav channel α-subunits contain four internally homologous domains (DI-IV). Each domain contains six transmembrane alpha helices (S1–S6) (Figure 2A). Helix S4 of each domain contains positively charged amino acid residues along one face of the helix. The movement of the S4 helices in response to changes in membrane potential is transmitted to helices S5 and S6 of each domain. This leads to the transient opening and subsequent inactivation of the channel pore [8,9]. High-resolution structures obtained by cryogenic electron microscopy (cryo-EM), for the heart-specific Nav1.5 α-subunit, the skeletal muscle channel Nav1.4 and the neuronal channels Nav1.2 and Nav1.7 show that the four domains surround the central pore with four-fold pseudosymmetry. Helices S1–S4 lie on the outer rim of the channel, with helices S5 and S6 from each domain forming the channel pore region [10,11,12,13,14]. This topology is highly conserved between Nav α-subunit isoforms, as illustrated by comparison of the Nav1.7 and Nav1.5 structures (Figure 2B).

### 1.2. The Nav Channel β-Subunits and Their Binding Sites on the α-Subunits

**Figure 3:**
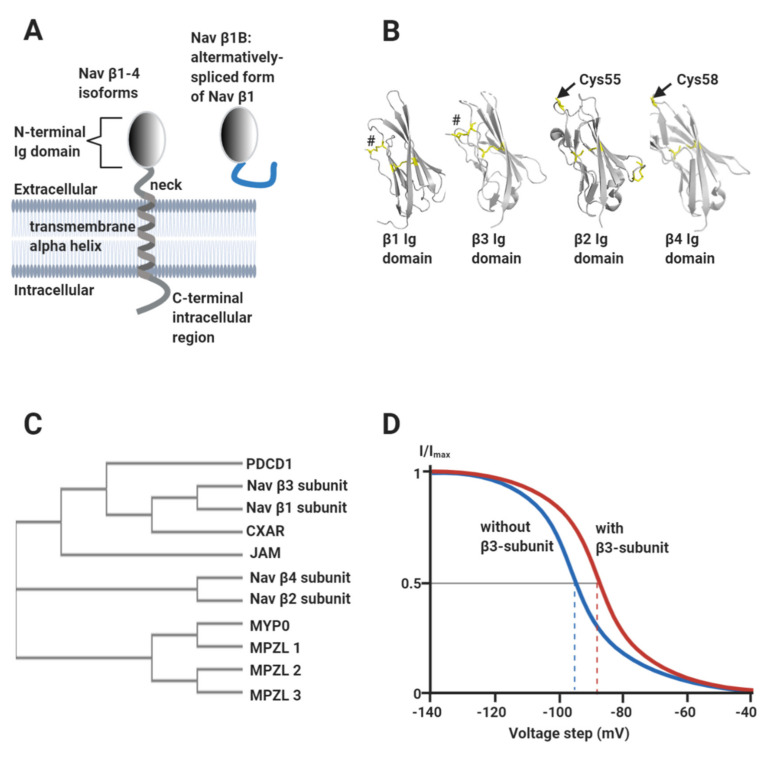
The Nav channel β-subunits. (**A**) Cartoon showing the common structural features of the β-subunits, including the alternatively spliced β1B isoform. (**B**) Atomic-resolution structures for the Ig domains of: β1 (PDB: 6JHI); β2, (PDB: 5FEB); β3 (PDB: 4L1D) and β4 (PDB: 5XAX). The separate disulphide bonds stabilising the N-terminal strands of β1 and β3 are labelled by hashtags and the free, exposed Cys residue on β2 (Cys55) and β4 (Cys58) are as indicated. (**C**) Phylogenetic analysis of Nav channel β-subunits, showing their relationship to members of the Ig domain-containing CAM protein family. PDCD1: programmed cell death protein 1 (https://www.uniprot.org/uniprot/Q15116); CXAR: Coxsackievirus and adenovirus receptor (https://www.uniprot.org/uniprot/P78310); JAM: junctional adhesion molecule 2 (https://www.uniprot.org/uniprot/P57087); MYP0: myelin protein P0 (https://www.uniprot.org/uniprot/P25189); MPZL1: myelin protein zero-like protein (https://www.uniprot.org/uniprot/O95297); MPZL2: myelin protein zero-like protein 2 (https://www.uniprot.org/uniprot/O60487) and MPZL3: myelin protein zero-like protein 3 (https://www.uniprot.org/uniprot/Q6UWV2). The phylogenetic tree was constructed using the ClustalW2 package (https://www.ebi.ac.uk/Tools/phylogeny/simple_phylogeny/). (**D**) Idealised inactivation curves of the Nav1.5 channel in the absence (blue) and the presence (red) of the β3-subunit. The β3-subunit induces a depolarising (rightward) shift of the V½ of inactivation, as indicated on the diagram by the dotted lines.

Vertebrate Nav channels are typically associated with one or more β-subunits (Mwt ~30–40 kDa). There are four homologous β-subunit genes (*SCN*1b-4b) encoding subunit proteins β1-β4 respectively. The β-subunits are type I transmembrane proteins consisting of a single extracellular N-terminal V-type immunoglobulin (Ig) domain, connected to a transmembrane alpha-helix by a flexible neck and terminating in a largely disordered intracellular C-terminal region (Figure 3A,B). An alternatively spliced form of β1, known as β1B, is also expressed in the heart. It consists of an Ig domain identical to that of β1, but lacks the transmembrane alpha-helix and is therefore secreted (Figure 3A) [15]. The β1- and β3-subunits show the closest sequence similarity to each other and are more distantly related to β2 and β4 (Figure 3C) [16,17]. The β-subunits have multiple effects on Nav channel gating behaviour that vary between individual β-subunit isoforms. In general terms however, they can increase the peak current density of Nav channels, probably by enhancing trafficking to the plasma membrane [2]. They also shift the voltage ranges over which Nav channel steady-state activation and/or inactivation occur, and in some cases enhance the rates of inactivation and recovery from inactivation [18]. As an illustrative example, the β3-subunit shifts the V_½_ for inactivation of Nav1.5 in a depolarising direction: i.e., the voltage at which half the channels are inactivated is displaced to a more positive value compared to the α-subunit alone (Figure 3D) [19,20,21,22]. For a cardiomyocyte with a resting potential of about -90 mV [2], this would act to increase the fraction of functional Nav1.5 channels available in the membrane [17].

**Figure 4:**
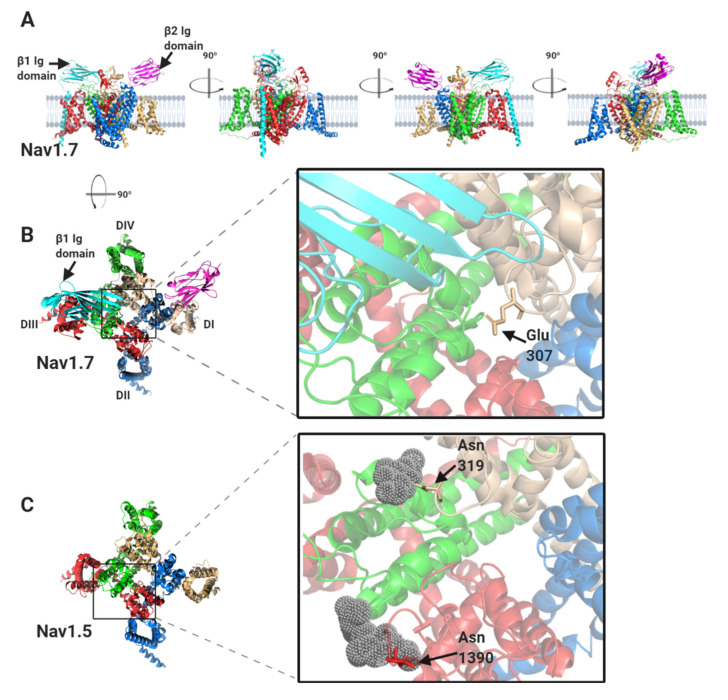

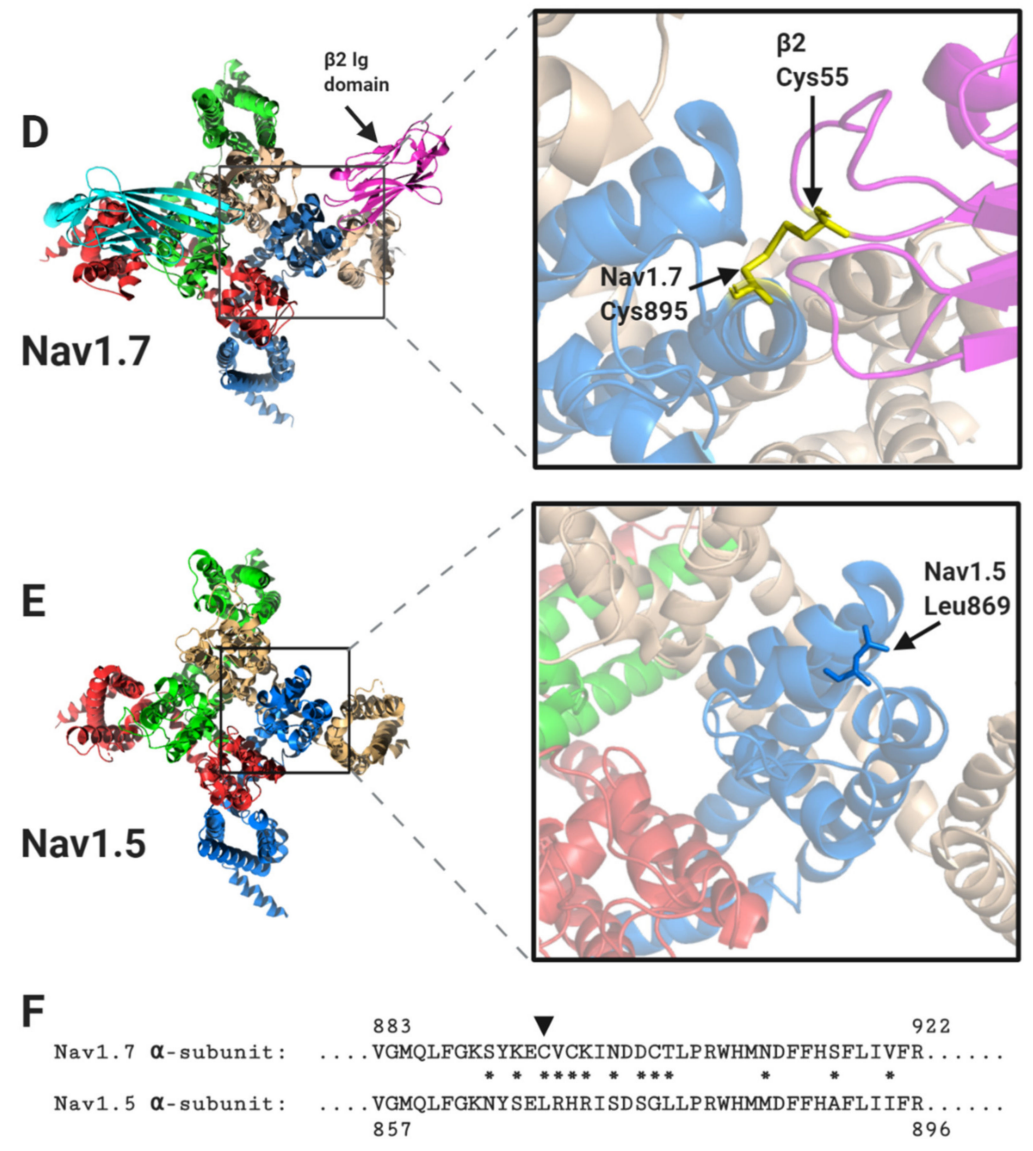
The binding sites for β1 and β2 on the Nav1.7 α-subunit and its comparison with Nav1.5. (**A**) Side views, each with 90° rotation, of the Nav1.7 α-subunit, with associated β1 and β2 -subunits. (**B**) Top view of the Nav1.7 α-subunit, with the β1 Ig domain binding site on the DI, DIII and DIV extracellular loops highlighted. (**C**) Top view of the equivalent region of Nav1.5 α-subunit. Resolved electron density corresponding to the N-linked sugar residues mentioned in the text are shown in grey dots. (**D**) Top view of the Nav1.7 α-subunit with the β2 Ig domain binding-site on DII extracellular loop highlighted. (**E**) Top view of the equivalent region of the Nav1.5 α-subunit. (**F**) Sequence alignment of human Nav1.7 and Nav1.5 α-subunits around the DII β2 binding-site. Amino acid differences between the two sequences are indicated by asterisks. The position of the Cys895 residue in Nav1.7, noted in the text, is indicated with an arrowhead.

The β1-subunit interaction site has been resolved at high resolution for Nav1.2, Nav1.4 and Nav1.7 α-subunits [10,11,12,13] and is illustrated for the case of Nav1.7 in Figure 4A,B. The β1-subunit Ig domain makes ionic and hydrogen-bond contacts with the DI, S5-P extracellular loop, the DIII, S1–S2 extracellular loop and the DIV, P-S6 extracellular loop regions (Figure 2 and Figure 4B). Surprisingly however, the Nav1.5 α-subunit structure has revealed some localised, but structurally significant differences between Nav1.5 and the other studied Nav channels [14]. In particular, the Nav1.7 Glu307 residue in the DI, S5-P extracellular loop, is changed in Nav1.5 to an asparagine residue, Asn319. This creates an N-linked glycosylation site that is not present in any other Nav channel isoform. In the Nav1.5 cryo-EM structure, there is electron density around Asn319 that is consistent with a complex N-linked glycan (Figure 4C). It should be noted that the electron density detected in the cryo-EM data only corresponds with two N-acetyl glucosamine residues of the core glycan. The remaining, diverse sugar moieties of the terminal branches are not resolved, presumably due to their inherent flexibility. Thus the N-linked glycan attached to Nav1.5, Asn319 extends further than the resolved electron density and would certainly be bulky enough to occlude the binding site for the β1 Ig domain [12]. Moreover, the specific orientation of a second N-linked glycan attached to Nav1.5 residue Asn1390 will probably also interfere with the binding of the β1 Ig domain (Figure 4C). Hence, it seems likely that in vivo, although β1 may still be associated with the Nav1.5 DIII voltage sensing domain via its transmembrane alpha-helix, its Ig domain will not be able to bind to the Nav1.5 α-subunit.

Based on biochemical and electrophysiological data, it is probable that the β3-subunit transmembrane alpha-helix also binds to Nav1.5 DIII voltage sensing domain [19,20,21]. Yet, there is evidence that it may bind closer to Nav1.5 DIII helix S3 rather than to the binding site for the β1 transmembrane region on the DIII helix S2 [19,21]. If so, then a given Nav1.5 α-subunit may be able to bind simultaneously to β1 and β3-subunits and there is indeed electrophysiological evidence to support this idea [21,23].

In contrast to β1 and β3, which bind to the α-subunit non-covalently, the β2-subunit binds to Nav1.7 covalently via a disulphide bond between a cysteine on the Ig domain (Cys55) and a corresponding cysteine (Cys895) on the α-subunit DII S5-P extracellular loop (Figure 4D) [12,24]. Neither the transmembrane alpha-helix nor the intracellular region of β2 is resolved in the published structure, indicating that both must be unconstrained in this purified complex [12]. As with β2, the β4-subunit Ig domain also contains a cysteine (Cys58) that can form a disulphide bond to the free cysteine on the α-subunit DII, S5-P site [25]. It is therefore presumed that the β2 and β4-subunit Ig domains covalently bind to the same or largely overlapping site on most Nav channel α-subunits [26,27]. Oddly however, the putative β2- and β4- subunit binding-site in Nav1.5 again shows important sequence differences from that on other Nav channels. Most notably, the residue equivalent to Cys895 of Nav1.7 is changed to leucine in Nav1.5 (Leu869) (Figure 4D–F). Since there are no other accessible free cysteines on the Nav1.5 extracellular surface, it will be impossible for either the β2- or the β4-subunit Ig domains to covalently bind Nav1.5 as they do to Nav1.7. Furthermore, the amino acid residues clustered around Nav1.7 Cys895 and which, in Nav1.7 provide additional contacts with the β2 Ig domain, are substantially different in Nav1.5 (Figure 4F).

Taken together, this evidence suggests that the Ig domains of all four β-subunits will be unable to bind to Nav1.5 directly, although the β-subunit transmembrane and intracellular regions may still do so. As a result, the Ig domains will be free to explore a greatly extended volume space above and around the Nav1.5 channel than β-subunits attached to most other Nav channels. What then are the likely functional consequences of this difference?

## 2. The Nav Channel β-Subunits as Cell-Adhesion Molecules

Phylogenetic analysis indicates that all four of the β-subunits are closely related to the myelin P0 family of cell-adhesion molecules (CAMs). Indeed, the β2 and β4-subunit sequences are more closely related to other myelin P0-like proteins, MPZL1-3, than they are to either β1- or β3-subunits [28,29,30] (Figure 3C). The β1-subunit can bind in *trans* both to itself and to other CAMs such as neurofascins and contactins [31]. The β2-subunit can bind in *trans* to extracellular laminin [32]. The β3-subunit can bind homophilically in *cis* [20,33] and heterophilically in *trans* with neurofascins [34]. The β4-subunits can bind homophilically both in *cis* and *trans* [35]. In all cases, these interactions occur via the Ig domains [36]. Hence, the Nav β-subunits can be considered CAMs in their own right; albeit with the additional property of Nav channel modulation.

### 2.1. The Nav1.5-Associated β1-Subunit as a CAM at the Intercalated Disc: Its Role in Ephaptic Conduction

**Figure 5:**
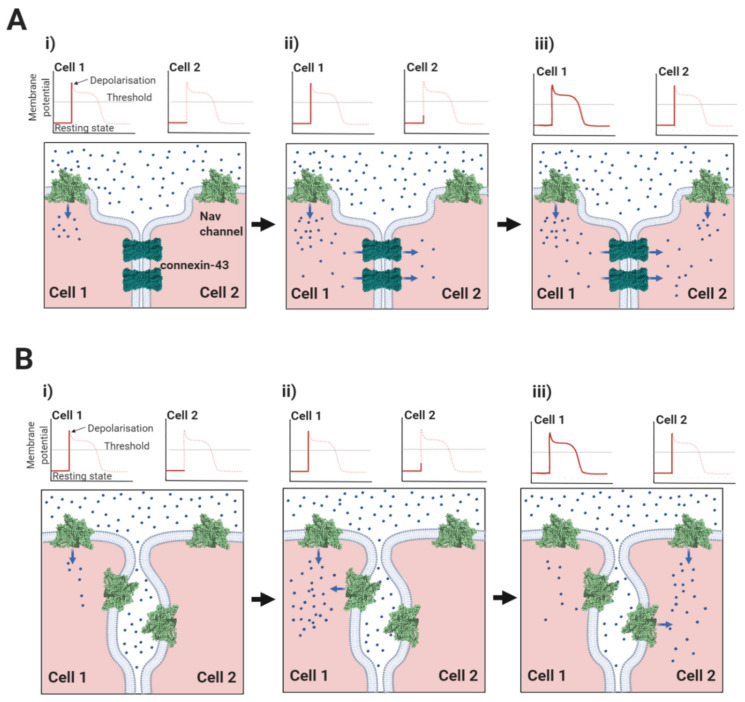
Comparison of electrotonic and ephaptic conduction mechanisms. (**Ai**); In electrotonic conduction, depolarisation of cell 1 by an action potential generates a membrane potential across the gap junction comprising connexin molecules. (**Aii**); The resulting current flow from active cell 1 to quiescent cell 2 causes its membrane to depolarise. (**Aiii**); When this changing membrane potential rises above the threshold for Nav channel opening, an action potential is initiated in cell 2 and the excitation wave is propagated. Note, the involvement of direct charge transfer between successive cells in this process, which involves an *ohmic* transfer of charge. (**Bi**); In ephaptic conduction, Nav channel excitation depolarises cell 1. (**Bii**); As a result, Nav channels in the perinexal membrane of cell 1 become depolarised and there is a removal of sodium ions (and hence a net removal of positive charge) from the ephaptic space separating cells 1 and 2. (**Biii**); The negative change in electrostatic potential within the restricted ephaptic space is enough to depolarise the transmembrane potential in cell 2, causing the activation of its Nav channels and propagation of the excitation wave. Note the absence of direct charge transfer between successive cells in this process, which involves an *electrostatic* transfer of charge. For clarity, only connexin-43 gap junction channels and Nav channels are shown, and in the action potential profiles, the Nav channel threshold has been displaced upwards.

The intercalated disc is formed from the juxtaposition of the sarcolemma membranes from adjacent cardiomyocytes and enables the cells to be mechanically and electrically coupled together [37]. It has a complex inter-digitated ‘plicate’ structure that ensures the inter-cellular connections are both close and continuous. Within the intercalated disc, there are distinct and structurally diverse domains each with their own specialised functions. For example, tight physical coupling at the desmosome and adherens junction help to transmit mechanical force between adjacent cells (Figure 1) [38,39]. Interspersed between these structures lie the gap junction plaques [40]. These domains contain a high local density of the protein connexin. Ventricular cardiomyocytes mostly express the connexin-43 isoform, although other isoforms are also present [41]. Connexins co-assemble on each membrane to form a symmetrical hexameric pore-containing hemichannel. Two hemichannels - one from each apposing membrane - associate in *trans* to form the functional gap junction and bind the two membranes together, so that they are no more than 2–4 nm apart, a distance that provides a tight inter-membrane seal [42]. Furthermore, a typical plaque contains several hundred closely packed gap junctions, ensuring the almost complete exclusion of other proteins from this region [41]. The central connexin pore is large enough to permit the passage of ions and small molecules [43]. Hence, the common view that electrical coupling between cardiomyocytes occurs predominantly by an electrotonic spreading mechanism in which charged ions flow passively from the cytoplasm of the ‘upstream’, depolarised cell to the cytoplasm of its neighbouring, ‘downstream’ quiescent cell via their shared gap junctions (Figure 5A) [44,45,46].

Surrounding and abutting the gap junction plaques lies the perinexus [47,48]. Unlike the gap junction, the perinexal membranes from adjacent cells do not interact directly, but enclose a restricted, inter-perinexal space, about 20 nm wide and extending away from the gap junction plaques for about 200 nm [48]. The perinexal membranes display a distinctive molecular composition. In particular, they contain a high local density of Nav1.5 channels that form multi-molecular two-dimensional clusters on the membrane surfaces [49]. This clustering may partly reflect an inherent tendency of Nav1.5 channels to self-associate [20]. But Nav1.5 channels are additionally stabilised by interactions with connexin-43 hemi-channels and with multi-modular cytosolic scaffolding proteins such as ankyrin-G, ZO-1 and 14-3-3 [50,51,52]. These interactions not only ensure the accumulation of Nav1.5, but they can also modify Nav1.5 activity. For example, ankyrin-G acts as a coordinating signalling hub, functionally connecting Nav1.5 gating with upstream kinase and phosphatase enzymes and down-stream cytoskeletal proteins [53]. In addition, 14-3-3 promotes co-operative gating behaviour between Nav1.5 α-subunits [54].

The clustering of Nav1.5 channels on the perinexal membranes, with the potential for synchronous depolarisation, ensure that activated channels on one membrane can withdraw enough sodium ions from the restricted inter-perinexal space, so that the reduced density of positive charge depolarises Nav channels on the perinexal membrane from the adjacent cell. This is the concept of ephaptic conduction, in which electrical communication between cardiomyocytes is achieved without the direct movement of sodium ions from one cell to the other (Figure 5B) [46,47,55,56,57,58]. Interestingly, perinexal Nav1.5 channels co-localise with the inwardly rectifying potassium channel Kir2.1 [59,60]. There is structural evidence that the C-terminal domains of Nav1.5 and Kir2.1 co-assemble on the cytoskeletal protein SAP97, and thereby form a functionally linked, macromolecular complex [61,62]. The Kir2.1 channel exhibits a relatively high potassium conductance when the cardiomyocyte is at the resting potential. Initially therefore, the movement of potassium ions into the perinexal space will buffer the local change in electrical potential when the Nav1.5 channels first begin to open. However, its inwardly rectifying property leads to a reduced conductance during the action potential plateau phase [63]. At this point, the entry of positively charged potassium ions into the perinexal space is minimised, just as the Nav1.5 channels are maximally depolarising and removing positively charged sodium ions. Hence, the Kir2.1 channel may act synergistically with Nav1.5 to finely regulate the rate and extent of ephaptic conduction [59].

Triggering ephaptic conduction would constitute a distinct non-canonical function of Nav channels in promoting cell-to-cell excitation, as opposed to the more familiar action potential conduction within the same cell. It could complement, or in some pathological circumstances, potentially replace electrotonic conduction mediated by gap-junctions [55,58]. Conversely, their disruption following pathological inflammatory or fibrotic change might compromise cell-cell conduction [64]. This could be in addition to disruptions in connexin-mediated cell-cell coupling [65]. It is in this context, that we suggest the unique structural features of Nav1.5 and its associated β-subunits should best be interpreted.

**Figure 6:**
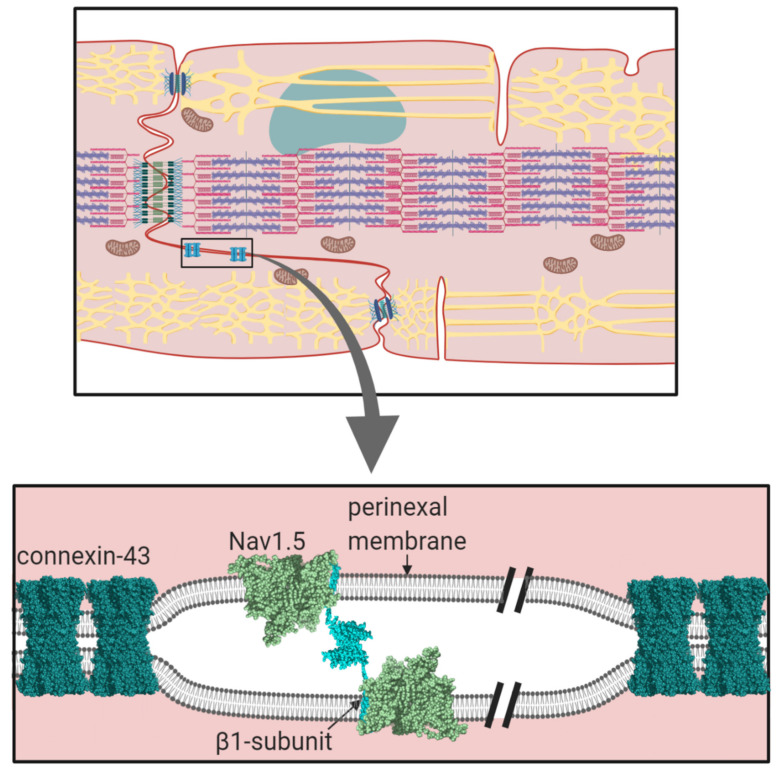
The proposed role for the β1-subunits in stabilising Nav1.5 channels on both apposing perinexal membranes, whilst also maintaining the necessary width between perinexal membranes to ensure efficient ephaptic conduction.

The perinexal Nav1.5 channels are associated with the β1-subunit. Recently, *Veeraraghavan, et al*., have shown that the perinexal membranes are associated with each other by *trans* homophilic binding between apposing β1 Ig domains [66]. However, if the β1 Ig domain is attached to Nav1.5 in the way that it is to Nav1.7, then it would only protrude about 4–5 nm above the membrane surface (Figure 4A). In that case, the Ig domains would not be able to cover the required 20 nm distance between perinexal membranes [47]. If on the other hand, the β1 Ig domain is not anchored onto the Nav1.5 α-subunit, then the two β1-subunits from apposing membranes are freer to extend their flexible necks and bridge this gap, forming *trans* cell-adhesion contacts (Figure 6). We suggest that the unique sequence differences in the DI and DIV extracellular loop domains of the Nav1.5 α-subunit compared to other Nav channels (Section 1.2, above) are specific adaptations that prevent Nav1.5 binding to the β1 Ig domain and so facilitate this specialised cell-adhesion behaviour of the β1-subunit. In consequence, the *trans*-mediated β1 Ig domain interactions help define the dimensions of the perinexal space by constraining its width to no more than about 20 nm. Mathematical modelling indicates that a gap of this order is optimum for ephaptic conduction [56].

**Figure 7:**
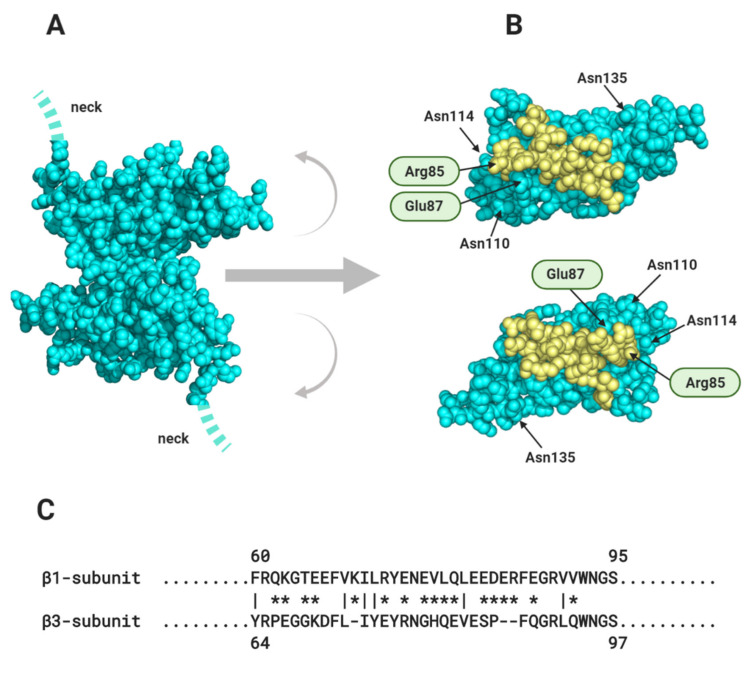
The proposed contact surface between the trans-interacting β1 Ig domains. (**A**) The antiparallel arrangement of β1 Ig domains. (**B**) the two β1 Ig domains ‘peeled back’ to reveal the contact surface proposed by [66]. The location of two Brugada mutations (Arg85 and Glu87), mentioned in the text are indicated. Potential N-linked glycosylation sites, Asn110, Asn114 and Asn135 are indicated. (**C**) Sequence alignment between β1 and β3 Ig domains within the proposed contact surface, showing low sequence identity. Non-conservative changes are shown with asterisks and conservative changes with vertical lines.

The two interacting β1 Ig domains form an extended antiparallel contact surface running between residues 66 and 86 (Figure 7A,B) [66,67]. In ventricular cardiomyocytes, a peptide mimetic of this binding site competitively inhibited *trans* binding between the two β1 Ig-domain. This induced a widening of the perinexal space, with a concomitant reduction in perinexal sodium current that precipitated arrhythmogenesis [66]. The clinical relevance of this work is supported by studies on patients with atrial fibrillation that show a correlation between arrhythmic severity and excessive widening of the atrial perinexal membranes [68]. Moreover, there are two independent *SCN*1b mutations within or close to this region that are associated with Brugada syndrome: a charge-neutralising Arg to His change at residue 85 and a charge-neutralising Glu to Gln mutation at residue 87 (Figure 7B) [69,70]. Taken together, these multiple structural, anatomical, pathological and experimental data sets not only support a role for ephaptic conduction between cardiomyocytes, but also implicate the structure, geometry and *trans*-binding behaviour of the β1-subunit as specific adaptations that are critical for this mechanism.

When the cardiomyocytes are at rest, the inter-perinexal space is largely enclosed, such that it is minimally influenced by its surrounding extracellular fluid. This too should increase the ability of activated Nav1.5 channels to remove enough positive charge to induce ephaptic conduction. Nevertheless, there must be some way for the electrolyte composition of the perinexal space to be reset between excitation events. This could be facilitated by geometrical changes produced by the cardiomyocyte contractions if they transiently enhance the accessibility of the perinexal space to the extracellular milieu. Real-time in vivo imaging of ventricular cardiomyocytes has identified surprisingly large flexing movements in the perinexus during the propagation of cell-to-cell contraction [71]. A consequence of these cellular and membrane movements will be the rhythmic stretching and relaxing of the *trans*-associated β1-subunits. Here, the disordered and spring-like neck region of the β1-subunit, connecting its rigid Ig domain to its rigid transmembrane alpha-helix might enable some strain-absorbing movement of the β1-subunit. However, as noted above, the β1 peptide mimetic can not only gain access to the perinexal space, it can also disrupt the *trans*-binding between β1 Ig domains [66]. This implies that the *trans* binding must—at least to some degree—be dynamic, perhaps reflecting a periodic dissociation and rebinding during the stretching cycle.

The β1-subunit can bind to cytosolic ankyrin-G and ankyrin-B via its intracellular region. But this interaction is abolished if a critical intracellular tyrosine residue, Y181, is phosphorylated by Fyn kinase [72,73]. In cardiomyocytes, the tyrosine-phosphorylated form of β1 is present only at the intercalated disc, where it interacts with Nav1.5 and the CAM cadherin, but does not bind ankyrin-G. The non-phosphorylated β1-subunit is present on the lateral membranes [74]. Of course, there could be many functional reasons for this pattern. For example, phosphorylation may be a targeting signal to direct β1-subunits to the intercalated disc [15]. Alternatively, the β1-subunit may be actively phosphorylated at the intercalated disc in response to changes in membrane stretching or other physiological signals. This would change the proportion of β1 that could connect to the cytoskeleton via ankyrin-G and thus modify cell-adhesion, perhaps even during a contraction cycle. One may speculate that the β1-mediated *trans* contacts could be further modulated via the alternatively spliced β1B isoform (Figure 3A) [15]. Since this isoform is secreted, it could in theory interrupt and fine-tune the *trans*-binding, if present in the perinexal space. There is as yet no direct evidence for this proposal, but a mutation in the β1B isoform is linked to Brugada syndrome [75].

### 2.2. Do Other Nav Channel β-Subunits Facilitate Ephaptic Conduction?

Although the β3 and β1-subunits show the closest overall sequence similarity (Figure 3C), there are localised sequence differences between them that could suggest functional specialisation. In particular, the sequence of the β1 Ig domain *trans* cell-adhesion binding site is not conserved in β3 (Figure 7C) [76] and the evidence that β3 can act as a homophilic *trans* CAM is mixed [76,77]. We therefore suggest that unlike β1, the β3-subunit may not play a major role in stabilising the perinexal space by *trans*-mediated cell-adhesion. On the other hand, when expressed alone in HEK293 cells, the β3-subunits bind homophilically in *cis*, using their Ig domains [19,20,33]. Super-resolution imaging experiments show that when co-expressed with Nav1.5, the β3-subunit affects the relative geometry between Nav1.5 channel α-subunits, possibly by promoting particular orientations of individual Nav1.5 α-subunit dimers within larger clusters [20]. This is consistent with a *cis*-mediated β3-subunit effect. The *cis*-interacting β3-subunit Ig domains could cross-link Nav1.5 channels within each perinexal membrane and thus contribute lateral stability to the channel clusters whilst the β1-subunit provides structural stability between apposing perinexal membranes.

Binding of the β2-subunit to Nav1.5 cannot be detected by immunoprecipitation, when tested in transfected HEK-cells [14]. Yet the β2-subunit can be immunoprecipitated together with Nav1.5 from heart tissue [78] and mutations in the β2 cytosolic region compromise the trafficking efficiency of Nav1.5 in cardiomyocytes [2]. Hence, additional cardiomyocyte-specific protein contacts are likely to be required to stabilise β2-binding to Nav1.5 in its normal physiological context. Interestingly, the Ig domains of the β1 and β2-subunits can interact in *trans*, but the association requires the presence of a sequence within the β2-subunit cytoplasmic region that contains a putative casein kinase II phosphorylation site [31]. This raises the possibility that *trans* heterophilic interactions between β1 and β2 Ig domains may occur across the perinexal space, yet be regulated by signal transduction events acting on the cytosolic face of the membranes [2].

**Figure 8:**
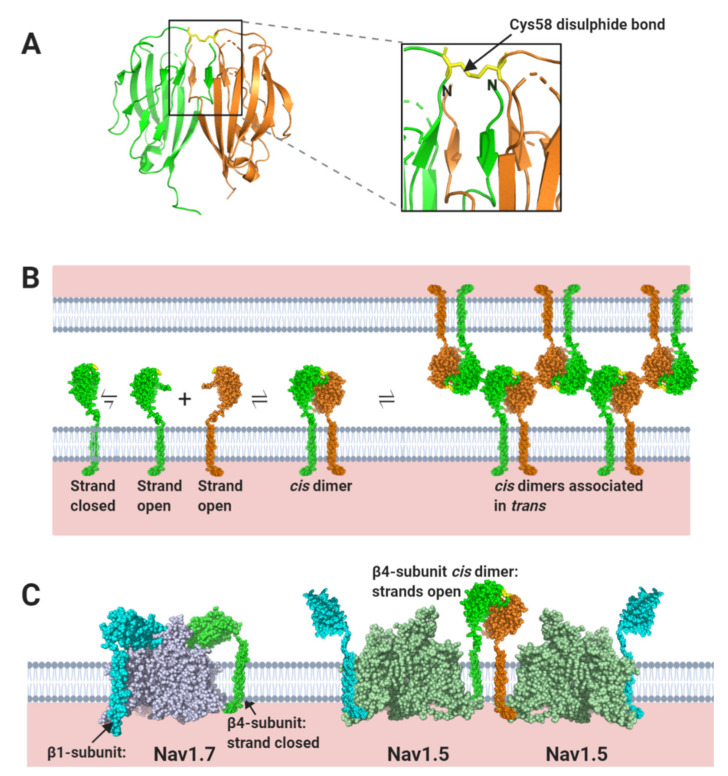
The β4-subunit Ig domain can associate in both *cis* and *trans*. (**A**) The disulphide-bonded, *cis*-interacting, dimeric β4-subunit Ig domain (PDB: 5XAW) with the Cys58 disulphide and the strand swap N-terminal regions highlighted. (**B**) Proposed model for *trans*-interacting, β4-subunit Ig domain *cis*-dimers, based on the model discussed in the text [79]. (**C**) Proposed association of the β4- and β1-subunits on Nav1.7 and Nav1.5 α-subunits. Note, to clarify the structural details in (A) and the cartoon models in (**B**) and (**C**), the two identical β4 Ig domains and β4-subunits are coloured differently, in green and orange.

The homophilic cell-adhesion contacts of the β4 Ig domain have been studied using crystallographic analysis, site-specific cross-linking with unnatural amino acids and cell-adhesion assays. The β4 Ig domain can be isolated as a dimer, stabilised by two striking features. Firstly, the presence of an inter-subunit disulphide bond between the Cys58 residue on each Ig domain. Secondly, a reciprocal strand-swap interaction, in which the first seven N-terminal amino-acid residues from one Ig domain interact with residues on the partner Ig domain (Figure 8A) [79]. N-terminal strand-swapping is a known feature of several *trans*-mediated CAMs that contain Ig domains, including cadherins in the adherens junctions and desmogleins in the desmosomes [80,81,82]. In the case of β4 however, the strand-swapped, disulphide-bonded Ig domain dimer is proposed to assemble on the same membrane in a *cis*-interaction. In this process, the N-terminal residues that undergo swapping must first undock from their binding site within the ‘closed’ monomer, to generate an ‘open’ monomer, which is then capable of *cis*-dimerisation (Figure 8B). A β4 Ig domain in which the Cys58 residue was changed to alanine, crystallised as a monomer in the asymmetric unit and the N-terminal strand was not resolved [26], suggesting that this conformation might correspond to the monomeric ‘open’ state. It has been further proposed that at the cell-surface, the β4 *cis* dimers, then interact in *trans* to promote cell-adhesion (Figure 8B) [35,79]. It is interesting to note that neither the β1-subunit, nor the β3-subunit Ig domains can form similar strand-swap dimers, because their N-terminal strands are covalently locked in place via an intramolecular disulphide bond [12,33,76] (Figure 3B).

These structural insights have an important implication. If β4 is paired with most Nav channel α-subunits, its Cys58 residue will form a disulphide bond with the free cysteine on the α-subunit DII S5-P extracellular loop site [25] (e.g., Nav1.7; Figure 4D and Figure 8C). But this would inhibit formation of the Cys58-mediated β4 *cis* dimer (Figure 8A,B). On the other hand, the Nav1.5 α-subunit lacks a free DII, S5-P cysteine. So, it is not possible for Nav1.5 to covalently bind the β4-subunit Ig domain (Section 1.2.). Nevertheless, the interaction between the β4-subunit and Nav1.5 is stable enough to be detected by immunoprecipitation when co-expressed in HEK293 cells [83]. Presumably, β4 must still bind to Nav1.5 via other sites such as the transmembrane alpha-helix and/or the cytoplasmic region. This will leave any Nav1.5-associated, β4-subunit Ig domains free to form disulphide-bonded, homophilic dimers (Figure 8C). Thus, potentially, the β4-subunit could facilitate not only *cis*- but also *trans*-mediated Nav1.5 cross-linking (Figure 8B,C). The overall dimensions of the β4 subunit are similar to β1 [17]. The β4 and β2-subunits can be detected very close to - but distinct from - the intercalated disc gap junctions [5], suggesting a likely perinexal location. It is tempting to speculate that the β4 (and probably β2) subunits could help stabilise both the width of the perinexal space and the clustering of Nav1.5 within this region by facilitating extensive *cis* and *trans*-mediated associations.

### 2.3. Nav Channels and β-Subunits on the Lateral Membrane: A Role in Mechanosensing?

A major fraction of the Nav1.5 channels on the lateral membrane are sequestered into lipid rafts and caveolae [84,85]. The caveolae are cholesterol-rich microdomains stabilised by the intrinsic scaffold membrane protein caveolin and the peripheral, cytosolic protein cavin [86]. Palmitoylation is commonly thought to act as a targeting signal that directs proteins into these structures [87]. Interestingly, the transmembrane alpha-helix of the β1 and β3-subunits (but not the β2 and β4-subunits) contains a juxtamembrane cysteine residue, located at the cytoplasmic interface that fits the consensus for a palmitoylation site [16,17]. Recent work confirms that β1 is indeed palmitoylated at this residue (Cys162). However, mutational abolition of the Cys162 residue does not prevent β1 targeting to detergent-resistant membrane fractions (assumed to correspond to lipid rafts), although it does reduce the steady-state level of the β1-subunit at the plasma membrane [88]. Within the caveolae, the Nav1.5 and β-subunit complexes are co-clustered with both Kir2.1 and the Kv4.2/4.3 channels responsible for the transient outward potassium current [84,89,90]. Surprisingly, the Nav channel β1-subunit binds to and regulates the Kv4.2/4.3 channels [91]. This opens the potential for mutual control of both channels via Nav β1 - especially so on the restricted and crowded caveolar membrane. The caveolae also sequester integrins, required for cell-adhesion to extracellular matrix proteins such as collagen and fibronectin, together with the integrin-activated signalling enzyme, Fyn kinase [86,92]. However, since the caveolae are highly invaginated, rosette-like clusters, it is not clear whether the sequestered integrins can bind extracellular matrix proteins under these conditions; neither is it clear whether the sequestered ion channels can be active [93]. Yet there is evidence that caveolin can regulate integrin activity [94] and in turn, both integrins and caveolin can modulate Nav1.5, Kir2.1 and Kv4.2/4.3 channels [95,96,97].

A possible explanation for these observations is that caveolae act as sensors for mechanical stress and respond dynamically to changes in cell deformation [98]. In particular, the cell stretching initiates a rapid dissociation of the cavin coat and a ‘flattening’ of the caveolae membrane. This mechanism increases the surface area of the plasma membrane and helps buffer against stretching forces [99,100]. A further consequence is that the integrins, ion channels and signal transduction enzymes are repartitioned into the plasma membrane, but probably remain as a macromolecular complex. This translocation is followed by an increased integrin-stimulated ERK signalling, which is caveolin-dependent [93]. Since ERK is a down-stream target of activated Fyn kinase [101], this suggests that the translocation of the integrin/ion channel complex into the wider plasma membrane can enhance integrin-dependent Fyn-kinase activation, perhaps by enabling greater access of the integrins to their extracellular matrix ligands. Activated Fyn kinase also phosphorylates Nav1.5, inducing a depolarising shift in the V_1/2_ of steady-state inactivation, thus increasing the fraction of functionally active Nav1.5 channels on the membrane [102]. Furthermore, Nav1.5 is a mechanosensor and the membrane flexing itself will affect channel gating. In particular, the Nav1.5 channel responds to physiological levels of mechanical membrane stress with a hyperpolarising shift to the V_1/2_ of steady-state inactivation [103,104]. Remarkably, the β1-subunit further amplifies this hyperpolarising shift [105]. The combination of increased Nav1.5 excitability via integrin-activated Fyn-kinase signalling, with the opposite mechanosensitive response of Nav1.5 and its β1-subunit may act to finely balance the need to integrate different cell-adhesion signals, whilst also protecting against excessive mechanical stress over a large voltage range. The β3-subunit enhances the mechano-induced acceleration of both activation and inactivation kinetics, but unlike β1, it does not affect the mechanosensitive shifts in steady-state parameters [105]. The structural explanation for these differences between β1 and β3-subunits and indeed their functional significance, is not clear.

The β-subunits exposed on the lateral membrane can interact with extracellular matrix proteins. For example, the β2-subunit can bind in *trans* to laminin, a known component of the heart extracellular matrix [32]. But there is relatively little β2-subunit exposed on the cardiomyocyte lateral membrane [5]. Another possibility is that β1-subunits exposed on the surface of the lateral membrane may interact homophilically with β1-subunits and other CAMs on adjacent cell types [31,34]. For example, during cardiac fibrosis, there is an enhanced proliferation of myofibroblasts that express several different Nav channels including Nav1.5, together with the β1-subunit [106]. Myofibroblasts can form aberrant electrical connections to the cardiomyocytes, which likely adds additional and potentially pro-arrhythmic membrane capacitance [107]. Whether the contacts between cardiomyocytes and myofibroblasts involve *trans*-cell adhesion contacts between β1-subunits remains to be determined.

### 2.4. Nav β-Subunits and Neuronal Channels in the T-Tubules

The neuronal Nav channels localised within the T-tubules are predominantly associated with the β1 and β3-subunits [5]. These Nav α-subunits all lack a glycosylation site in their DI, S5-P extracellular loop, equivalent to Asn319 in Nav1.5 [9]. Hence, it is likely that the β1 and β3 Ig domains will bind onto these Nav α-subunits in a manner more like Nav1.7 than Nav1.5 (Figure 4A,B). As the T-tubule width is significantly greater than 20 nm (and is often more than 100 nm) [108], any direct *trans*-mediated binding of β1 Ig domains between opposite membrane faces of the T-tubules is unlikely. There is evidence that CAMs such as laminin can enter the lumen of large-diameter T-tubules [109]. So, cell-adhesion-type functions for T-tubular β-subunits cannot be ruled out. In mice, deletion of the *SCN*1b gene perturbs the interaction between T-tubular Nav channels and NCX complexes, with a consequent dysregulation of calcium homeostasis, suggesting a structural role to facilitate excitation-contraction coupling [110].

## 3. Conclusions and Unsolved Problems

On the cardiomyocyte membranes, the Nav channels form heterogeneous, multi-component macromolecular clusters, rather than remain as isolated molecules [111]. There is no necessary requirement for every Nav α-subunit to have an identical stoichiometry with any associated β-subunit [17]. Examples from other ion-channels show that the behaviour of membrane-bound clusters can change depending on variations in subunit ratios [112]. In such assemblies, individual protein components can have more than one function, depending on the physiological context. Although originally identified by their direct effects on channel gating, it is now clear that the Nav β-subunits extend their functions to include cell-adhesion and mechano-sensing and in doing so, raise further questions:

### 3.1. Evolutionary Relationship between Nav β-Subunits and Other CAMs

The Ig domain superfamily has deep evolutionary roots that pre-date the divergence of vertebrate and invertebrate lineages [113]. Yet the Nav channel β-subunits have only been discovered in vertebrate genomes, where they cluster together with members of the Ig domain-containing CAM family [30]. This close evolutionary relationship raises the intriguing possibility that homologues such as the MPZL1-3 group of proteins (Figure 3C), might act as additional Nav channel modulators. At least some of these proteins are expressed in heart tissue [114].

Although lacking β-subunits, invertebrate Nav channels do possess associated proteins that modulate gating and trafficking behaviour of their Nav channels. The best characterised are members of the TipE family [115]. However, these proteins show no sequence or structural similarity to vertebrate β-subunits and must have independently evolved their Nav channel-modulating behaviour. It will be interesting to see whether the TipE proteins can act as CAMs, or whether cell-adhesion is a unique feature of the vertebrate β-subunits.

### 3.2. The Biophysics of Nav β-Subunit Cell-Adhesion

We currently lack a quantitative understanding of the *trans*-mediated binding events facilitated by the β-subunits. For example, it would be interesting to know if the contacts between individual β1 Ig domains at the perinexus are strong and stable or individually weak enough to dissociate and rebind rapidly. The latter case might be more likely given the dynamic nature of membrane movements at the intercalated disc during the contraction, relaxation cycle [71]. The application of new biophysical techniques such as atomic force microscopy and traction force microscopy [116], combined with more traditional biochemical and molecular genetic techniques will be needed to address these questions.

### 3.3. The Role of N-Linked Glycosylation

Membrane proteins are generally N-linked glycosylated, with complex, branching sugar residues, often tipped with sialic acid moieties [117]. The role of N-linked glycosylation in the trafficking of Nav channels, including Nav1.5 - is well-established [118]. There is also evidence that the negatively charged sialic acids on N-linked glycans of Nav channel α and β-subunits can modulate channel gating [119]. In addition, the relatively large and bulky N-linked glycans can potentially modulate the strength and even the possibility of protein-protein interactions occurring. A good example is described above for the case of the β1 Ig domain binding to Nav1.5, and the likely role of the glycosylated Asn319 residue in preventing binding of the β1 Ig domain (Section 1.2, Figure 4C). Another example is in the model proposed for β1- *trans* cell-adhesion. Here, the putative *trans*-binding motif on the β1 Ig domain surface is surrounded by four of its five potential N-linked glycosylation sites (Section 2.1, Figure 7B). Could the strength of this interaction be fine-tuned by for example, developmentally regulated changes in the nature and extent of N-linked glycosylation?

### 3.4. Ephaptic Conduction in the Heart and Elsewhere

In cardiomyocytes, ephaptic conduction occurs in close association with gap junction structures mediating electrotonic conduction (Section 2.1, Figure 5), suggesting that both processes occur to relative extents, that might vary under different conditions [120]. It is likely that there are other biological situations where the necessary conditions for ephaptic conduction apply. Potential examples include the repetitive firing that occur in neuroendocrine supraoptic nucleus neurones [121,122] and the escape reflex triggered by activation of the goldfish Mauthner neurone [123]. Interestingly, the R85H mutation in the β1 Ig domain, that compromises ephaptic conduction between cardiomyocytes [66] (Section 2.1, Figure 7B), also predisposes to epilepsy [124], perhaps hinting at a similar role in neurones.

### 3.5. Clinical Implications

Assuming that electrical signalling between cardiomyocytes occurs both by electrotonic and ephaptic mechanisms, then a drug that inhibits the *trans*-mediated cell-adhesion between perinexal β1-subunits might reduce the signal propagation through cardiac muscle, whilst not completely preventing it. This could potentially reduce triggering of post-infarct arrythmias [125]. Conversely, drugs that stabilise these interactions could be useful as a treatment for other forms of arrythmias such as Brugada syndrome in which re-entrant arrhythmia results from a conduction slowing substrate [66]. It might also be possible to target specific β-subunit signalling pathways, for example the phosphorylation of the β1-subunit cytoplasmic region [126]. These are quite speculative, yet potentially attractive hypotheses that require further investigations. More broadly, the increasing emphasis on the cell-adhesion roles of Nav β-subunits in both healthy and pathological states, offers a more balanced perspective on these proteins and could open completely new avenues for therapy.
